# Feasibility and preliminary efficacy of tantalum components in the management of acetabular reconstruction following periacetabular oncologic resection in primary malignancies

**DOI:** 10.1186/s40001-022-00777-x

**Published:** 2022-08-17

**Authors:** Pengfei Zan, Xiaojun Ma, Hongsheng Wang, Zhengdong Cai, Jiakang Shen, Wei Sun

**Affiliations:** 1 Department of Orthopedics, Shanghai General Hospital, School of Medicine, Shanghai Jiao Tong University, Shanghai, 200080 China; 2 Department of Orthopedics, Jintan People’s Hospital, Changzhou, Jiangsu Province China

**Keywords:** Periacetabular oncologic resection, Tantalum components, Acetabular reconstruction, Prosthetic survivorship

## Abstract

**Background:**

The aim of the study was to investigate the feasibility and preliminary efficacy of tantalum components utility in the reconstruction of acetabular defects following periacetabular oncologic resection of primary malignancies.

**Methods:**

We prospectively collected a consecutive of 15 cases that were treated with tantalum components for acetabular reconstruction after periacetabular oncologic resection from January 2018 to December 2018. The cohort included 8 male and 7 female patients, with a mean age of 47.6 years (range, 33 to 67 years). Pathology types: chondrosarcoma (*n* = 9), malignant bone giant cell tumor (*n* = 3) and osteosarcoma (*n* = 3). Clinical outcomes, functional and radiographic results were recorded in detail for analysis.

**Results:**

Patients received planned oncologic resection and tantalum components reconstruction without casualty; they were followed up with a mean of 39.7 months (35–45 months). The mean operation time was 4.0 h (3.0–6.0 h), and the mean blood loss was 1260 ml (800–2200 ml). Functional outcomes were assessed by MSTS-93 scale, with an average of 21.8 (12.0–26.0 scores), among which 3 cases were excellent, 11 were good and 1 was fair. The mean Harris Hip Score was 79.1scores (46.0–92.0 scores) at 1-year follow-up postoperatively. 3(3/15, 20.0%) cases experienced postoperative complications: 2 cases with hip dislocation received closed reduction under general anesthesia and were fixed with hip joint abduction braces for 6 weeks; one case had a superficial infection and received debridement with a delayed wound healing. Oncologic prognosis: one case relapsed at 8-month follow-up and received hemi-pelvic amputation; and another osteosarcoma patient experienced relapse with pulmonary metastasis and received further chemotherapy. No prosthetic loosening, displacement or fracture occurred during the follow-up period.

**Conclusion:**

Preliminary results suggested that the use of tantalum components in the management of acetabular reconstruction following periacetabular oncologic resection provided reasonable improvement on functional outcomes and early stability of the prostheses. Porous tantalum components are conducive to bony ingrowth, which is a potential alternative to various existing reconstruction techniques to achieve better functional outcomes.

## Introduction

Treatment of acetabular defects after periacetabular oncologic resection demands proper implant selection and is a technical challenge for orthopedic surgeons given the complicated anatomies and biomechanics of the pelvic ring [1, 2]. Various biological and non-biological reconstructive options have been described in recent literatures, which include allograft prosthetic composites [3], modular metallic hemi-pelvic endoprostheses [4], modular saddle-shaped prostheses [5], custom-made endoprostheses [6], and autologous femoral head implantation [7].

Acetabular defects after periacetabular oncologic resection can be comparable to a revision of total hip arthroplasty (THA), in which porous tantalum components have been successfully used in achieving a mechanically stable hip structure [8]. The porous tantalum components possess several advantages over conventional cementless cups: higher percent volume of porosity, freely communicating pores, a bony-similar flexibility, higher coefficient of friction and biocompatibility for osseointegration and bony ingrowth [9]. Excellent results have been achieved with modular porous tantalum shells in revision THAs, with or without the use of tantalum augments or buttresses in a mid-term to long-term follow-up period [10, 11]. Therefore, the superiority of tantalum components could theoretically solve the weaknesses of the hemi-pelvic endoprostheses in other scenarios. To the best of our knowledge, there are rare reports on the use of tantalum components in the acetabular reconstruction following periacetabular oncologic resection of primary malignancies.

Therefore, a cohort study was conducted that included a consecutive of 15 patients in our single center who were recently treated with tantalum components for acetabular reconstruction after periacetabular oncologic resection. The purpose of the study was to determine the feasibility and preliminary efficacy of this reconstructive technique on clinical outcomes, functional and radiographic results as well as complications.

## Methods

### Patients’ selection

Ethical approval was obtained prior to conducting this cohort study by the local institutional review board. Patients diagnosed with primary periacetabular malignancies and received acetabular reconstruction with tantalum components following oncologic resections from January 2018 to December 2018 were collected. The following inclusion criteria were applied: (1) primary periacetabular malignancies that are resectable based on the principles of complete resection; (2) the defects can be reconstructed using tantalum components and total hip replacement; and (3) a postoperative period follow-up of more than 2 years. Exclusion criteria included: (1) tumors that had undergone previous curettage, resection, and cryosurgery or radiofrequency ablation; (2) malignancies with multiple metastases; (3) metastatic malignancies. A consecutive of 15 cases was identified for analysis according to the above inclusion and exclusion criteria, of whom 8 cases were male and 7 cases were female patients with a mean age of 47.6 years (33.0–67.0 years).

The main manifestation of the malignancies was pain and limping of the hip. All patients underwent preoperative X-ray, computed tomography (CT) and magnetic resonance imaging (MRI) examinations to confirm the presence of the tumors; positron emission tomography (PET)-CT were obtained for all cases to assess for any distant metastases. Preoperative biopsy was performed in each case to confirm pathology types, of which 9 (60.0%) were chondrosarcoma, 3 (20.0%) malignant bone giant cell tumor (MBGCT) and 3 (20.0%) osteosarcoma. Based on the Enneking pelvic surgical zone, 3 cases were located in zone I + II, 1 case in zone II, 6 cases in zone II + III and the other 5 case sin zone I + II + III.

### Surgical techniques

After confirming the diagnoses of the malignancies by imaging and pathology, each patient received a preoperative angiography examination to pinpoint the blood supply and followed with a vascular embolization. All surgeries were performed under general anesthesia with patients lying on their unaffected side. An extended iliofemoral Smith-Petersen (S-P) approach, a modified Kocher–Langenbeck (K–L) or the combination was utilized to perform the procedures based on the Enneking surgical zone of each tumor. Surgical techniques varied among cases given the nature of different oncologic resections and individual reconstructions. However, there are some basic principles: foremost, a clear oncologic surgical margin was initially guaranteed in all cases, the surrounding muscles were preserved as much as possible to ensure adequate soft tissue coverage and to reduce the risk of early dislocation and wound complications; after oncologic resection, the off-the-shelf tantalum components were used to reconstruct the acetabulum on structural and biological remaining bone; an acetabular cup was placed with the target of 40°of inclination and approximately 15°to 20°of anteversion, and modular fluted tapered stem or revision stems was finally inserted to optimize the limb length and offset, meshes were wrapped to the prostheses for soft tissue adherence, the tantalum components and prostheses used in this series are modular manufactured by Zimmer company.

**Fig.1:**
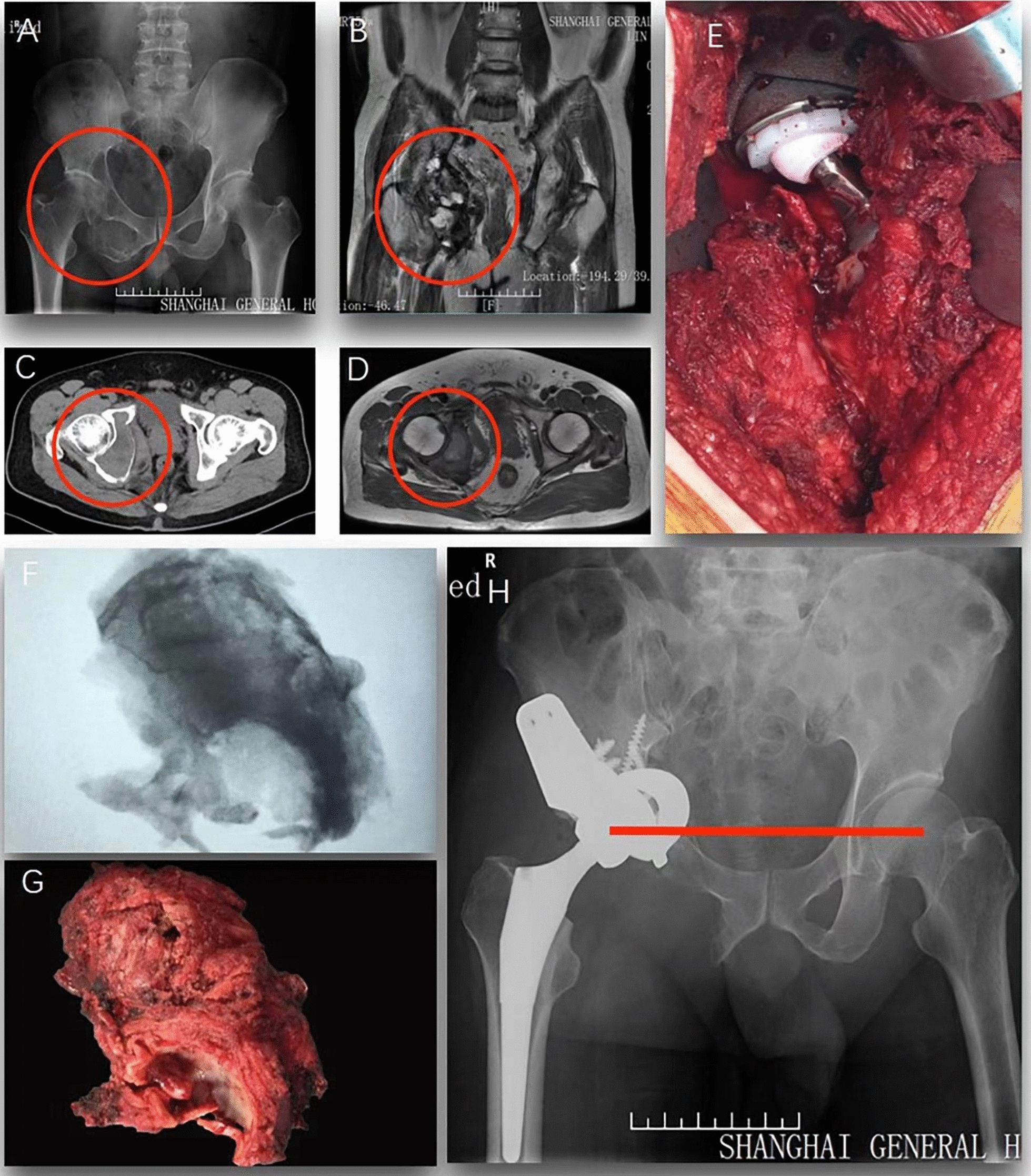
A male patient, diagnosed with pelvic chondrosarcoma (Enneking Zone II + III, type1 defects). **A**: preoperative X-ray indicates a tumor located at the right side of acetabulum and ischium; **B**: coronal MRI T-2 image indicates an enhanced signal at acetabulum and ischium; **C** and **D**: preoperative cross-section CT and MRI T-1 images indicate the tumor involved acetabulum; **E**: intraoperative images shows the tumor was resected and reconstructed with tantalum components and total hip arthroplasty; **F** and **G**: the tumor was completely removed (gross specimen and X-ray image); **H**: X-ray at 1-year postoperatively indicates a stable internal fixation and alignment

**Fig.2:**
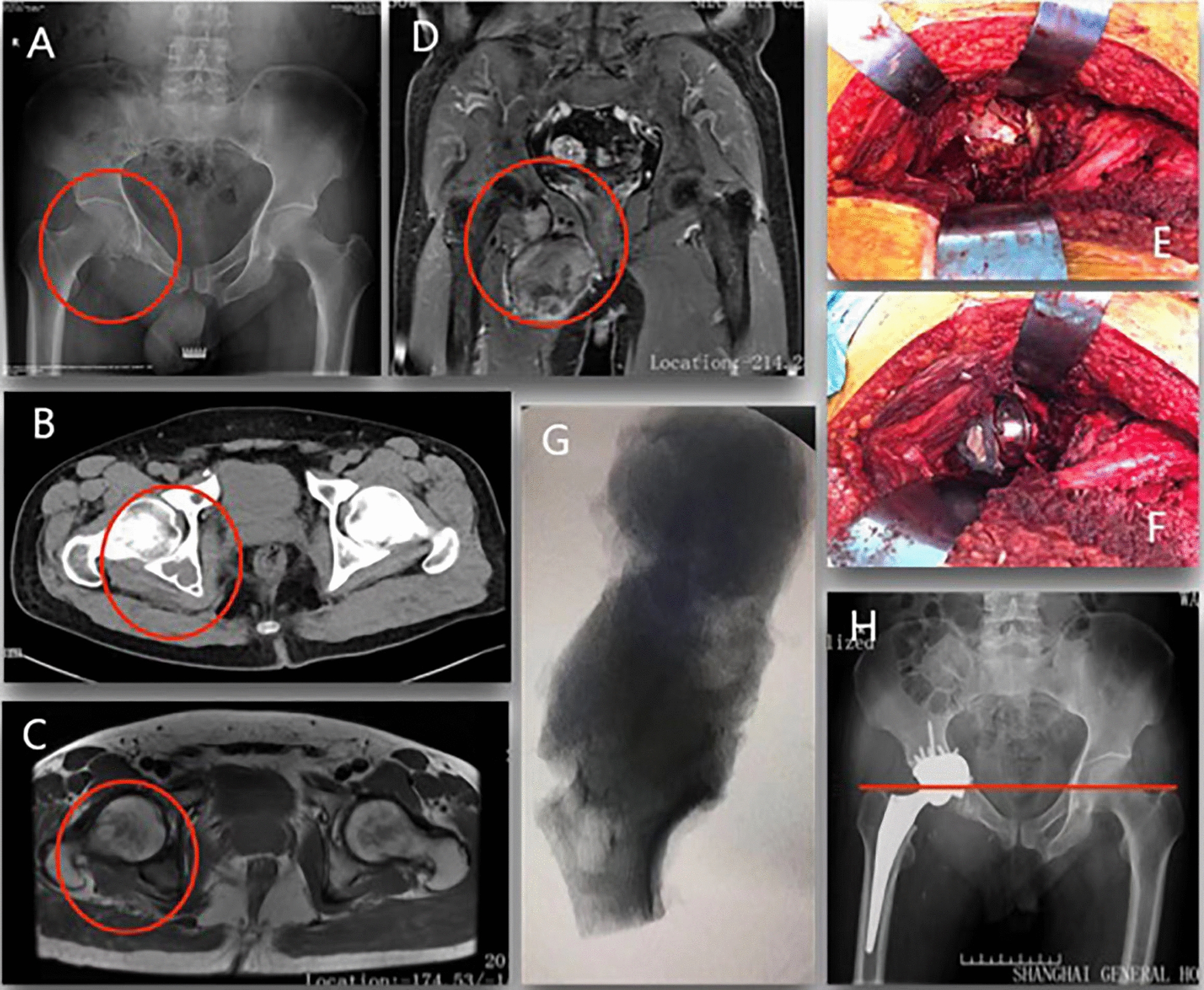
A male patient, diagnosed with pelvic MBGCT (Enneking Zone II + III, type2 defects). **A**: preoperative X-ray indicates tumor located at the right side of acetabulum; **B**: preoperative CT scan indicates the tumor involved the lower part of the acetabular; **C**, **D**: preoperative MR images indicate huge soft tissue mass involved acetabulum and ischium; **E**: intraoperative image showed the tumor was completely removed with dome of the acetabular reserved; **F**: intraoperative image showed the acetabular was reconstructed with tantalum component and bone cement; **G**: X-ray image of the resected tumor; **H**: pelvic X-ray at 1-year postoperatively indicates a stable internal fixation and alignment

**Fig.3:**
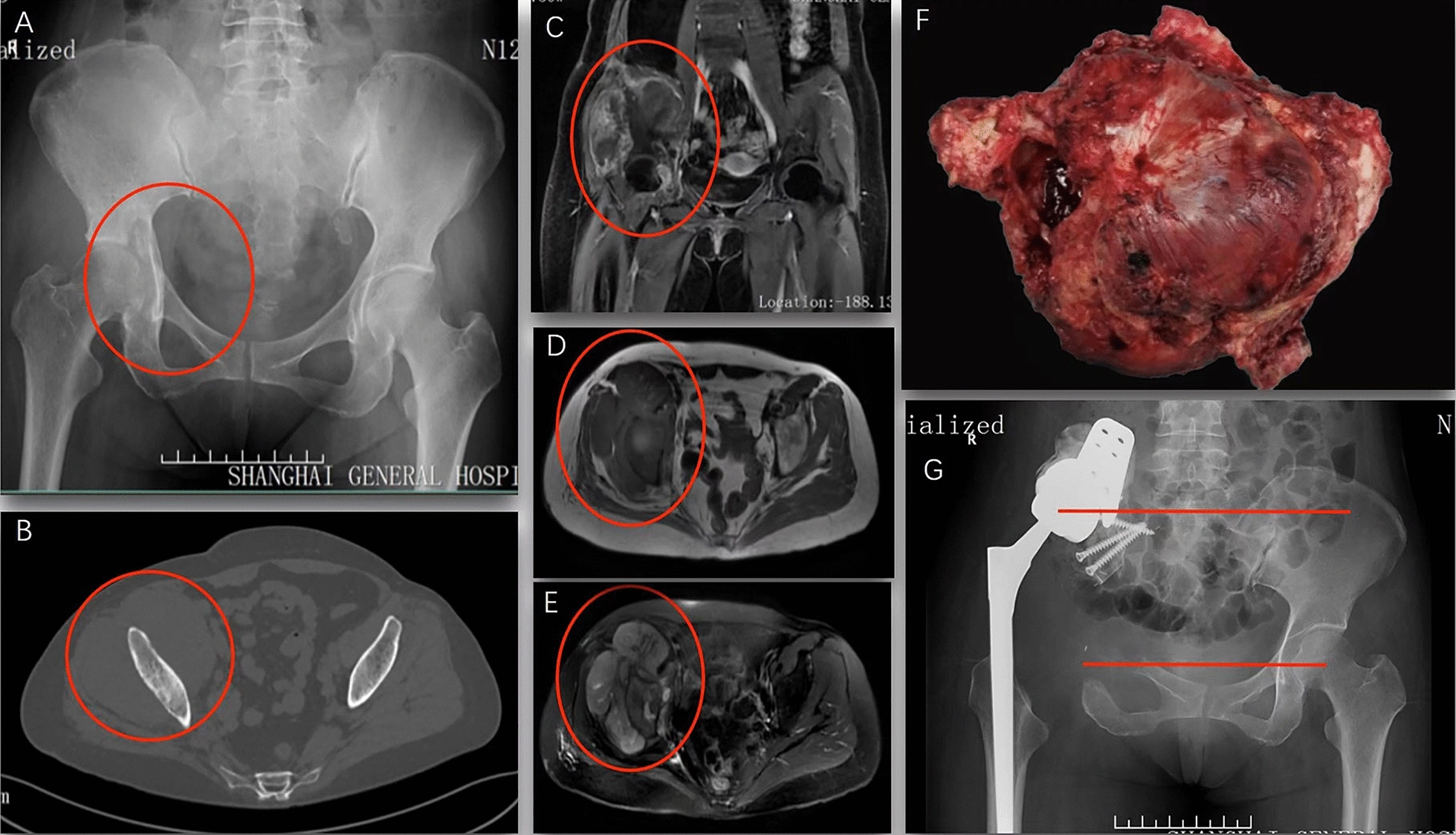
A female patient, diagnosed with pelvic chondrosarcoma (Enneking Zone I + II + III, type3 defects). **A**: preoperative X-ray indicates tumor located at the right side of acetabulum; **B**: preoperative CT scan indicates a huge soft tissue mass involved ilium; **C**, **D** and **E**: preoperative MR images indicate huge soft tissue mass involved acetabulum and ilium; **F**: the tumor was completely removed (gross specimen); **G**: pelvic X-ray at 1-year postoperatively indicates the upper-shifted rotation center, a stable internal fixation and alignment

Acetabular reconstruction was individually performed based on the acetabular defects after oncologic resection. Defects and reconstructions could be divided into three types: (1) pelvic continuity with partial acetabular defects, which correlates with the Enneking pelvic surgical zone II or II + III. For this type, the cartilage in the acetabulum was reamed by acetabular reamers; thereafter an appropriate hemispherical tantalum component was placed and fixed to the host bone for reconstructing the roundness of the new acetabulum. A biological acetabular cup was inserted; the gaps between the cup and tantalum component were implanted with the autologous femoral head fragments. A case is shown in Fig. 1. (2) Pelvic discontinuity with partial dome of the acetabulum, which correlates with the Enneking pelvic surgical zone II or II + III. In this type, partial cartilage in the acetabular dome was reamed, followed with a rectangular tantalum component fixing to the inner and outer plate of the ilium. A biological acetabular cup was placed on the dome and fixed with screws; the gaps were filled with bone fragments. The tantalum component and acetabular cup were finally coated with polymethylmethacrylate (PMMA) cement to increase the initial stability. A case is shown in Fig. 2. (3) Complete acetabular deficiency, which correlates with Enneking pelvic surgical zone I + II or I + II + III. In this type, the acetabular reconstructive technique was similar to the above courses. The differences were that the rotation center needs to be moved upwards and a longer femoral side prosthesis with a bigger offset was a necessity to increase the tension of soft tissue and to maintain the joint stability. A case is presented in Fig. 3.

### Postoperative management

Drains were removed in cases with a drain volume of less than 100 ml in 24 h or on postoperative day 3. Antibiotic prophylaxis cefuroxime (1.5 g) was intravenously administrated half an hour before operation and another equal dose was followed if the operation exceeds 3 h, in the postoperative period, prophylactic antibiotics were stopped within 72 h and would be delayed or elevated to high-grade antibiotics if there is any sign of inflammation. Rehabilitation therapists were involved; patients were instructed to practice the muscle strength of lower extremity in beds and were mobilized to sit up beside the beds 1 week postoperatively, and they were allowed to stand up assisted with double crutches at 4 weeks postoperatively.

### Follow-up outcomes

Patients received outpatient follow-up at 6 weeks, 3 months, 6 months post-operation and every 6 months afterwards. Pelvic X-ray was routinely performed as well as CT if necessary to evaluate the prosthetic situation. Chest CT was also obtained to exclude pulmonary metastases. Patients received a systemic isotope bone scan every 6 months after surgery to assess local control of the malignancy. Complications were recorded in detail if there were any.

Functional outcomes were assessed using the 1993 Musculoskeletal Tumor Society Scale (MSTS-93) [12] and the Harris Hip Scores (HHS) [13]. MSTS-93 scale constitutes 6 items, with a total of 30 points. A score of 80–100% is excellent, 60–79% is good, 40–59% is fair and less than 40% is poor. HHS contains 4 items (pain, function, deformity and range of motion). The score ranges from 0 to 100 with a higher score representing less dysfunction and better outcomes. MSTS-93 scale and HHS score were obtained pre-operatively and were re-evaluated as primary outcomes at 1-year follow-up.

## Results

Demographic data are shown in Table. 1. Patients were followed up for at least 2 years with a mean of 39.7 months (35.0–45.0 months). They received successful surgical procedures with no deaths at the most recent follow-up. The average operation time was 4.0 h, and the mean blood loss was 1260 ml (800–2200 ml).

**Table 1:** Demographic characteristics of the included patients

No.	Sex	Age (years)	Side	Diagnosis	Enneking zone	Prosthesis type	Follow-up (months)	Complications	MSTS-93 score	HHS score
1	M	42	Right	Chondrosarcoma	II + III	Cup, buttress, augments	42	None	26	88
2	F	36	Right	Chondrosarcoma	II	Cup, buttress	41	None	25	86
3	F	47	Left	Chondrosarcoma	I + II	Cup, buttress	37	None	22	85
4	M	51	Left	Osteosarcoma	I + II + III	Cup, buttress, augments	38	None	21	84
5	F	44	Right	Chondrosarcoma	I + II + III	Cup, buttress, augments	45	Delayed wound healing	23	82
6	M	47	Left	MBGCT	II + III	Cup, augments	40	None	23	76
7	M	53	Left	Osteosarcoma	I + II + III	Cup, buttress, augments	43	Dislocation	19	60
8	F	48	Right	Chondrosarcoma	II + III	Cup, augments	38	None	26	91
9	M	64	Left	Chondrosarcoma	I + II	Cup, buttress	38	None	25	88
10	F	55	Right	Chondrosarcoma	II + III	Cup, augments	37	Relapse, hemi-pelvic amputation	12	46
11	F	33	Right	Chondrosarcoma	I + II	Cup, buttress	44	None	26	92
12	M	56	Right	MBGCT	II + III	Cup-cage, augments	42	None	23	76
13	M	36	Left	Osteosarcoma	I + II + III	Cup, buttress, augments	39	Pulmonary metastasis	18	73
14	M	52	Left	MBGCT	I + II + III	Cup, buttress, augments	36	Dislocation	18	78
15	F	45	Right	Chondrosarcoma	II + III	Cup, augments	35	None	20	81

*M* male, *F* female, *MBGCT* malignant bone giant cell tumor, *MSTS-93* 1993 Musculoskeletal Tumor Society Scale, *HHS* Harris Hip Scores, MSTS-93 score and HSS score were evaluated 1-year postoperatively

During the follow-up period, a patient with chondrosarcoma experienced local relapse at 8 months postoperatively and was treated with hemi-pelvic amputation. This patient was followed for 37 months and had no further recurrence or metastasis. One patient with osteosarcoma had pulmonary metastasis at 6 months postoperatively and received additional chemotherapy, who was followed up recently at 39 months postoperatively and manifested with no sign of progression. Other patients did not show any evidence of tumor relapse or distant metastasis at the most recent follow-up.

Functional scoring was assessed at postoperative 1 year using MSTS-93 scale system, with an average score of 21.8 scores. Among them 3 cases were graded as excellent, 11 were as good, and 1 case was as fair. The patient graded as fair experienced a local relapse and had hemi-pelvic amputation. An average score of 79.1 at 1-year post-operation by HHS system for the evaluation of functional outcomes improved greatly compared to those assessed before operation.

A total of 3 (20.0%) cases had postoperative complications. 2 patients experienced hip dislocations during the hospitalization, who were treated with closed reduction under general anesthesia and followed by an abduction brace fixation for 6 weeks, neither had further dislocation. 1 case of chondrosarcoma had continuous faint yellow wound drainage and diagnosed with superficial infection, who was followed by the treatment of debridement and antibiotics and had a delayed wound healing. No other complications were detected in this cohort at the most recent follow-up. Radiographic evaluations showed no evidence of prosthetic loosening, displacement or fracture during the follow-up period.

## Discussion

Reliable reconstruction of acetabular defects after periacetabular oncologic resection of primary malignancies has historically been challenging whereas with limited options for selection. In the present study, we utilized porous tantalum components to reconstruct these acetabular defects in a consecutive of 15 patients. Here, we demonstrated a technical feasibility in the intraoperative manipulation and a reliable fixation in the setting of acetabular deficiency. In addition, a substantial improvement in functional outcomes with relatively low complications was achieved at an early postoperative follow-up period.

In 1974, Schöllner and colleagues [14] presented the first case of pelvic prosthetic reconstruction as an alternative to hemipelvectomy; and thereafter, the Harrington technique with threaded pins and a cemented acetabular implant was widely used for acetabular reconstruction after periacetabular oncologic resections [15]. Even though the design and materials of the prostheses are constantly improved, the long-term survival of these non-biological reconstructions remains low and prosthesis-related complications did not decrease [16, 17]. With the evolving of medical techniques in patients with primary periacetabular malignancies, life expectancy has increased. As such, achieving a biological reconstruction is vitally important [18]. The advent of porous tantalum components had achieved excellent clinical outcomes in the reconstruction of acetabular defects in revised THA procedures [9, 11, 19, 20]. Joglekar et al. [21] demonstrated reliable and durable constructions in patients who received a THA with a porous tantalum component following prior pelvic radiation. The success of these tantalum components has spurred a great interest in the oncologic arena. Houdek et al. [22] retrospectively reviewed a total of 58 patients treated with a tantalum acetabular implant and THA to reconstruct the metastatic neoplastic periacetabular lesions. At mid-term follow-up, it showed that the porous tantalum components can successfully serve as a stable, well-fixed and durable construct, without any case of mechanical failure. In primary periacetabular malignancies, a more precise resection can be achieved under meticulous preoperative planning and principles of complete resection. As such, part of the acetabulum or ilium can be preserved. The utilization of these porous tantalum components for reconstruction should theoretically prolong the prosthetic survivorship, especially for those who are younger and have prolonged life expectancies for curative purposes [7, 18]. In our cohorts, procedures suggested that the malignancies were settled under the principles of complete resection, thus the reconstructive techniques were not related with oncologic prognosis but with therapeutic expectancy as well as functional rehabilitation. The porous tantalum components provided with reliable fixation, thus, allowed patients to perform earlier rehabilitation exercises to achieve better functional outcomes.

From a clinical standpoint, we applied MSTS-93 scale and HHS scoring system to evaluate the postoperative 1-year functional outcomes. Early results showed an average MSTS-93 scale score of 21.8 with an excellent and good rate of 93.3%, and an average HHS of 79.1 in the current cohorts. The overall functional results suggested that they were not inferior to those after hemi-pelvic prosthesis reconstruction [6, 23, 24]. We came across two cases of dislocation that were treated with closed reduction under general anesthesia and were fixed with abduction braces. This is consistent with other studies in the literature describing dislocation as a common problem in patients receiving acetabular reconstruction after periacetabular oncologic resection [17, 25]. Abdel et al. [26] reported 3 cases of early dislocation among 10 patients who underwent oncologic acetabular reconstruction, of whom 2 were revised with constrained liner. We assumed that the hip dislocation was due to the destroyed hip abductors during oncologic resections. Whether an increased level of constraint or delayed sitting and walking exercises were necessary remain to be an issue mandating further investigation.

After a pelvic tumor resection, individual reconstruction should be comprehensively considered on the basis of a patient’s age, tumor location, functional requirements and life expectancy [27]. Houdek et al. [28] compared saddle prosthesis with tantalum THA and found that in selected cases, reconstruction with tantalum THA is of benefit with higher MSTS scores. Wang et al. [29] retrospectively reviewed 25 patients who received pelvic tumor resection and hemi-pelvic endoprosthesis reconstruction, results indicated that total complication rate was 56.0%, periprosthetic infections and aseptic loosening were most common. Guo et al. [30] developed modular hemi-pelvic prostheses to reconstruct acetabular defects; early results indicated an acceptable outcomes. Whereas, another Guo team reported mid-term outcomes indicated an increased rate of major complications requiring surgical intervention and a decrease in functional outcomes, which were drawbacks commonly observed in the hemi-pelvic prostheses reconstruction [31]. In the current cohorts, hemi-pelvic prostheses were notably not an optimal choice for those who were younger with potential long life expectancies. In contrast, biological reconstruction might be a better consideration in the context of a complete oncologic resection [7, 22]. In certain cases whose partial acetabulum or ilium was retained, the porous tantalum component is an alternative solution for the reconstruction of hip functions with additional autologous femoral head segments utilized for implantation. Early radiographic results indicated stable fixations with no evidence of prosthetic loosening, displacement or fracture at the most recent follow-up. Improved long-term prosthetic survivorship would be anticipated by performing this biological reconstruction in the following follow-up period.

Several limitations of the present study need to be addressed. Foremost, this study represented a very small cohort. This was because periacetabular malignancies are rare and only a few patients are indicated for this reconstruction. Second, the follow-up period was relatively short to evaluate the oncologic prognosis and long-term prosthetic survivorship. Lastly, we were unable to compare the performed technique to hemi-pelvic prosthesis reconstruction or others, resulting in a difficulty to verify whether our technique is superior to the alternatives. Despite limitations, we prospectively collected 15 consecutive patients and demonstrated a feasible and reliable technique to manage acetabular reconstruction after periacetabular tumor resections. We are looking forward to the long-term follow-up results of this cohort to further evaluate the oncologic prognosis and confirm longer prosthetic survival.

## Conclusion

The porous tantalum components represent a surgical solution to manage acetabular reconstruction after periacetabular tumor resection of primary malignancies. Preliminary results indicated that this reconstructive technique provided a reliable fixation with substantially improved functional outcomes and acceptable complication rates during early postoperative follow-up. The porous tantalum components are conducive to bony ingrowth, thus can be expected to improve long-term survival of the prosthesis.

## Data Availability

The dataset supporting the conclusions of this article is available on request—please contact the corresponding author.

## References

[1] Hasenauer MD, Paprosky WG, Sheth NP (2018). Treatment options for chronic pelvic discontinuity. J Clin Orthop Trauma.

[2] Shao QD, Yan X, Sun JY (2015). Internal hemipelvectomy with reconstruction for primary pelvic neoplasm: a systematic review. ANZ J Surg.

[3] Donati D, Di Bella C, Frisoni T (2011). Alloprosthetic composite is a suitable reconstruction after periacetabular tumor resection. Clin OrthopRelat Res.

[4] Zhang Y, Tang X, Ji T (2018). Is a Modular Pedicle-hemipelvic Endoprosthesis Durable at Short Term in Patients Undergoing Enneking Type I + II Tumor Resections With or Without Sacroiliac Involvement?. Clin OrthopRelat Res.

[5] Jansen JA, van de Sande MA, Dijkstra PD (2013). Poor long-term clinical results of saddle prosthesis after resection of periacetabular tumors. Clin OrthopRelat Res.

[6] Sun W, Li J, Li Q (2011). Clinical effectiveness of hemipelvic reconstruction using computer-aided custom-made prostheses after resection of malignant pelvic tumors. J Arthroplasty.

[7] Sun W, Zan P, Ma X (2019). Surgical resection and reconstructive techniques using autologous femoral head bone-grafting in treating partial acetabular defects arising from primary pelvic malignant tumors. BMC Cancer.

[8] Issack PS (2013). Use of porous tantalum for acetabular reconstruction in revision hip arthroplasty. J Bone Joint Surg Am.

[9] Theil C, Schmidt-Braekling T, Gosheger G (2019). A single centre study of 41 cases on the use of porous tantalum metal implants in acetabular revision surgery. BMC MusculoskeletDisord.

[10] Brown NM, Hellman M, Haughom BH (2014). Acetabular distraction: an alternative approach to pelvic discontinuity in failed total hip replacement. Bone Joint J.

[11] Konan S, Duncan CP, Masri BA (2016). Porous tantalum uncemented acetabular components in revision total hip arthroplasty: a minimum 10-year clinical, radiological and quality of life outcome study. Bone Joint J.

[12] Enneking WF, Dunham W, Gebhardt MC (1993). A system for the functional evaluation of reconstructive procedures after surgical treatment of tumors of the musculoskeletal system. Clin OrthopRelat Res.

[13] Nilsdotter A, Bremander A (2011). Measures of hip function and symptoms: Harris Hip Score (HHS), Hip Disability and Osteoarthritis Outcome Score (HOOS), Oxford Hip Score (OHS), Lequesne Index of Severity for Osteoarthritis of the Hip (LISOH), and American Academy of Orthopedic Surgeons (AAOS) Hip and Knee Questionnaire. Arthritis Care Res (Hoboken).

[14] Schöllner D, Ruck W (1974). Proceedings: Pelvic prosthesis–an alternative to hemipelvectomy in tumor patients. Z OrthopIhreGrenzgeb.

[15] Harrington KD (1981). The management of acetabular insufficiency secondary to metastatic malignant disease. J Bone Joint Surg Am.

[16] Brown TS, Salib CG, Rose PS (2018). Reconstruction of the hip after resection of periacetabular oncological lesions: a systematic review. Bone Joint J.

[17] Angelini A, Calabrò T, Pala E (2015). Resection and reconstruction of pelvic bone tumors. Orthopedics.

[18] Khan FA, Rose PS, Yanagisawa M (2012). Surgical technique: Porous tantalum reconstruction for destructive nonprimary periacetabular tumors. Clin OrthopRelat Res.

[19] Löchel J, Janz V, Hipfl C (2019). Reconstruction of acetabular defects with porous tantalum shells and augments in revision total hip arthroplasty at 10-year follow-up. Bone Joint J.

[20] Long WJ, Noiseux NO, Mabry TM (2015). Uncemented porous tantalum acetabular components: early follow-up and failures in 599 revision total hip arthroplasties. Iowa Orthop J.

[21] Joglekar SB, Rose PS, Lewallen DG (2012). Tantalum acetabular cups provide secure fixation in THA after pelvic irradiation at minimum 5-year follow-up. Clin OrthopRelat Res.

[22] Houdek MT, Abdel MP, Perry KI (2019). Outcome of patients treated with porous tantalum acetabular implants for neoplastic periacetabular lesions. J Am AcadOrthop Surg.

[23] Bus MP, Szafranski A, Sellevold S (2017). LUMiC^®^ endoprosthetic reconstruction after periacetabular tumor resection: short-term results. Clin OrthopRelat Res.

[24] Liang H, Ji T, Zhang Y (2017). Reconstruction with 3D-printed pelvic endoprostheses after resection of a pelvic tumour. Bone Joint J.

[25] Puchner SE, Funovics PT, Hipfl C (2014). Incidence and management of hip dislocation in tumour patients with a modular prosthesis of the proximal femur. Int Orthop.

[26] Abdel MP, von Roth P, Perry KI (2017). Early Results of Acetabular Reconstruction After Wide Periacetabular Oncologic Resection. J Bone Joint Surg Am.

[27] Tan TJ, Aljefri AM, Clarkson PW (2015). Imaging of limb salvage surgery and pelvic reconstruction following resection of malignant bone tumours. Eur J Radiol.

[28] Houdek MT, Wunder JS, Abdel MP (2021). Comparison of reconstructive techniques after acetabular resection for pelvic chondrosarcoma. Bone Joint J.

[29] Wang B, Zou C, Hu X (2019). Reconstruction with a novel combined hemipelvic endoprosthesis after resection of periacetabular tumors involving the sacroiliac joint: a report of 25 consecutive cases. BMC Cancer.

[30] Guo W, Li D, Tang X, Yang Y, Ji T (2007). Reconstruction with modular hemipelvic prostheses for periacetabular tumor. Clin Orthop Relat Res.

[31] Guo Z, Li J, Pei GX, Li XD, Wang Z (2010). Pelvic reconstruction with a combined hemipelvic prostheses after resection of primary malignant tumor. Surg Oncol.

